# Guided-wave manipulation in SIW H-plane horn antenna by combining phase correction and holographic-based leakage

**DOI:** 10.1038/s41598-022-15123-8

**Published:** 2022-07-04

**Authors:** Ali Araghi, Mohsen Khalily, Okan Yurduseven, Pei Xiao, Rahim Tafazolli

**Affiliations:** 1 Home of 5G & 6G Innovation Centres, Institute for Communication Systems (ICS), University of Surrey, Guildford, GU2 7XH UK; 2 School of Electronics, Electrical Engineering and Computer Science, Centre for Wireless Innovation, Queen’s University Belfast, Belfast, BT3 9DT UK

**Keywords:** Electrical and electronic engineering, Electronics, photonics and device physics

## Abstract

A hybrid technique is proposed to manipulate the field distribution in a substrate integrated waveguide (SIW) H-plane horn to enhance its radiation characteristics. The technique comprises two cascaded steps to govern the guided waves in the structure. The first step is to correct the phase of fields and form a quasi-uniform distribution in the flare section so that the gain increases and side-lobe-level (SLL) decreases. This is obtained by loading the structure with a novel modulated metal-via lens. Field expansion on the radiating aperture of the SIW H-plane horn generates backward surface waves on both broad walls which increases the backlobe. In the second step, these backward surface waves are recycled and directed forward with the aid of holography theory. This is achieved by adding a couple of dielectric slabs with holographic-based patterns of metallic strips on both broad walls. With this step, the backlobe is reduced and the endfire gain is further increased. Using the proposed technique, the structure is designed and fabricated to operate at $f=30$ GHz which simultaneously improves the measured values of gain to 11.65 dBi, H-plane SLL to $-\,17.94$ dB, and front-to-back ratio to 17.02 dB.

## Introduction

Substrate integrated waveguide (SIW) is a technology that can be used to build a variety of guided-wave structures^1,2^. SIW H-plane horn antenna (first introduced by Li et al.^3^) has attracted considerable attention due to its inherent properties including low-profile, ease of fabrication, and compatibility with planar printed circuit boards (PCBs). In comparison with conventional air-filled horn antennas, the field distribution inside the flares of SIW horns is considerably distorted which can decrease the gain and degrade the overall radiation characteristics. Several methods have been presented in the literature to enhance the radiation performance of the SIW H-plane horn antennas. These methods can be divided into three main categories as below.

In the first category, a component will be placed in front of the aperture to control the emanating electromagnetic (EM) fields^4–6^. This technique typically enlarges the dimensions of the structure. As an example, it is proposed to apply dielectric lenses with elliptical and rectangular geometry to achieve a higher gain with narrow beamwidths^4^. However, the size of structure is almost doubled. A similar challenge occurs in Ref.^5,6^ where the SIW H-plane horn is loaded by an air-via perforated dielectric slab and a dielectric slab with rectangular metal patch respectively.

The second category contains those techniques that will modify the geometrical characteristics of the structure in the flare section, mostly for tailoring the phase distribution^7–11^. For instance in Ref.^7^, a pair of slots are employed in the top and bottom metallization of the flare to reduce the back-lobe; the position of slots are specified by a try-and-error technique. An iterative method is also utilized in Ref.^8^ where a genetic algorithm is applied to pixelate both broad walls of the SIW horn to control the field distribution in the front panel of antenna. Conformal transformation optics is applied to gradually eliminate the phase error within the flare to enhance the gain of an H-plane horn by maximum 2.4 dBi^9^. Another method is to apply post metalized via-holes inside the horn carefully to make an in-phase wavefront in a same transverse line^10^. It is also proposed to load the flare section by a set of equally distributed metal pins across the center line of broad walls to form a slow-wave structure; this minimizes the phase difference between the center and the edges of the flare which ultimately improves the gain^11^. As the presented geometry can not be easily manufactured by printed circuit techniques, the prototype is embodied as a metal-only structure, making an air-filled H-plane horn. Air medium for propagation inside the guided-wave structure is also reported in Refs.^12–14^ which enhances the efficiency and gain, but brings fabrication complexity.

Finally, the third category refers to the combination of the first two categories^15–18^. By introducing the gap-SIW inside the flare section, the phase distribution is modified and then tapered-ladder transition is employed in front of the aperture to increase the gain^15^. But the method requires software optimizations to obtain phase correction and impedance matching simultaneously. With the aid of a dipole array, reflector nails, and a pair of transversal slots inside the flare section, a complex and fragile structure is obtained to improve the antenna gain^16^. Parallel transition with a narrow slot around the opening aperture are applied to enhance the antenna performance which makes the thickness of structure increased more than three times^17^. Optimizing the horn shape, followed by employing an array of transition printed patches on the same SIW substrate improves the radiation characteristics of the conventional SIW H-plane horn^18^.

Metamaterial lenses have been suggested to be applied inside the conventional pyramidal horn antennas to correct the phase^19,20^. But to the best of our knowledge no tantamount has been reported so far for the SIW H-plane horns.

In this paper, we propose a novel and practical hybrid technique for regulating the EM fields to achieve an enhanced radiation pattern out of the aperture in the SIW H-plane horn antenna. The idea is based on cascading two different techniques where the radiation characteristics improve by each subsequently. An original method is introduced to design a modulated metal-via lens with a systematic approach to realize its geometrical properties and location. This makes the first EM-manipulating component and improves the radiation characteristics. Then, based on the holography technique^21^, a new method is presented and developed to design the second EM-manipulating component to enhance the radiation properties even further. Using the proposed hybrid technique, we managed to simultaneously improve the gain, side-lobe-level (SLL), and front-to-back ratio (F/B) of a SIW H-plane horn antenna at the center frequency of 30 GHz. It is worth mentioning that the structure remains low-profile, even after loading it with the second EM-manipulating components. Last but not least, the method will not add any complexity to the fabrication process of conventional SIW structures.

## Overview on the proposed hybrid technique

**Figure 1:**
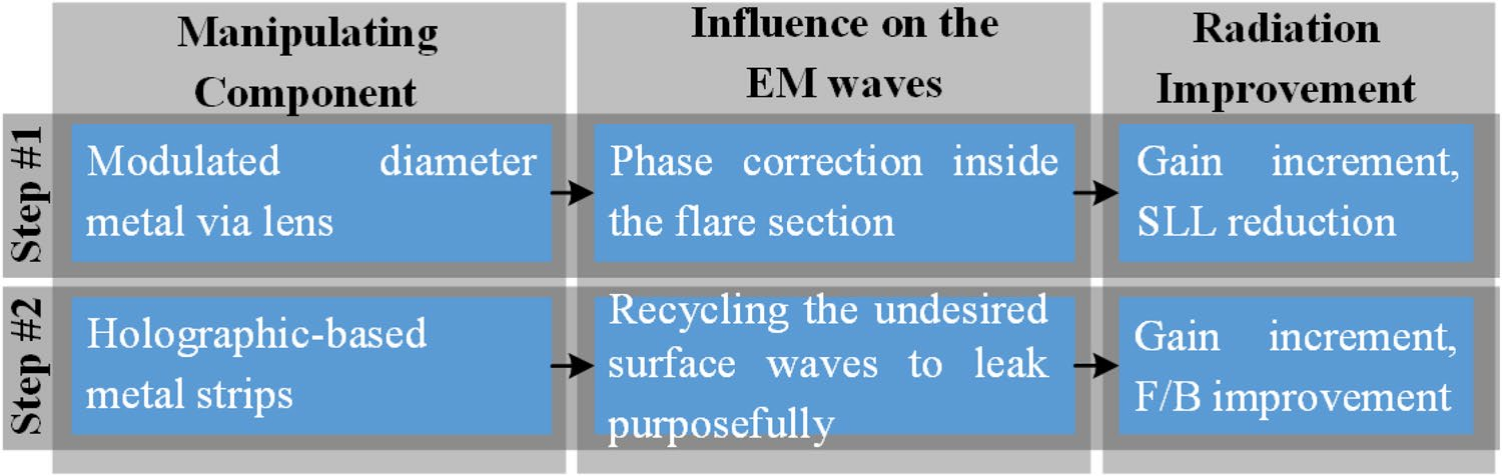
The proposed hybrid technique to enhance the radiation characteristics of the SIW H-plane horn antenna.

Uniform phase distribution on a radiating aperture results in proper illumination which can increase the gain^22^. On the other hand, a surface-wave packet toward any direction other than the desired one typically results in increasing the side/back lobes. Considering these principles, the proposed hybrid technique splits into two subsequent steps as shown in Fig. 1 and summarized below:

*Step #1* this step is based on regulating the transmission response of metalized via holes, posted in the substrate, to mimic a desired phase pattern on a transversal cut inside the flare section. This loading method can be classified into the second category of those mentioned in the previous section so that the size of the modified structure will be intact after applying the technique. The loading leads to a quasi-uniform phase distribution at the radiating aperture of the SIW H-plane horn antenna which increases the gain and decreases the SLL. Then, by studying the effective aperture of the modified structure, we manage to make the physical size of the structure smaller.

*Step #2* the end-fire radiating aperture of the SIW H-plane horns causes backward surface waves on both broad walls, increasing the backlobe. With the aid of holography theory, our idea is to recycle these undesired surface waves to make them leak purposefully for further improvement of gain and increasing the F/B. This can be fulfilled by attaching two dielectric slabs with holographic-based printed metallic strips on the broad walls of the modified H-plane horn.

In the following sections, we present the proposed hybrid technique in details.

## Modulated metal-via lens: proposed method to regulate the electromagnetic fields (step #1)

The fundamental requirement of designing a SIW with metalic vias of diameter *d* and period *p* is

1 $$\begin{aligned} d<\frac{\lambda _g}{5} \quad p<2d, \end{aligned}$$

where $\lambda _g$ is the guided wavelength of the dominant mode^23^.

**Figure 2:**
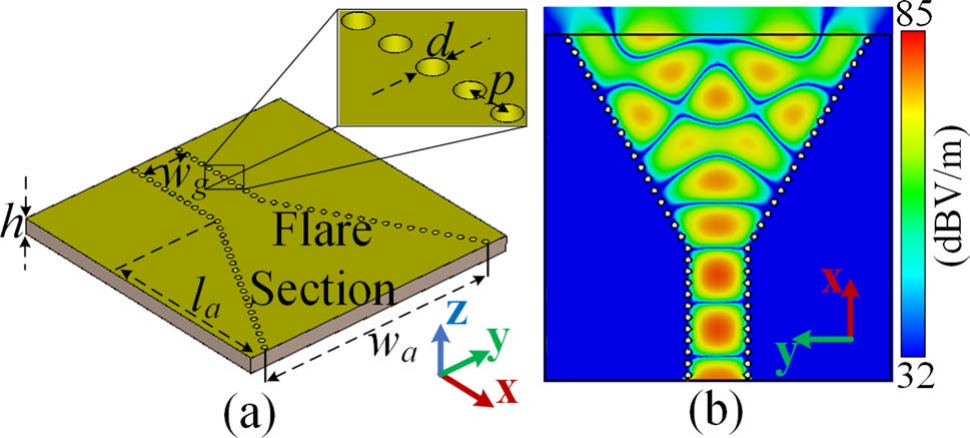
Conventional SIW H-plane horn antenna. (**a**) The geometry, (**b**) the simulated |E-field| distribution pattern at 30 GHz.

Considering an operating frequency of $f=30$ GHz, a conventional SIW H-plane horn antenna and the corresponding |E-field| distribution pattern (simulated in CST MWS) are shown in Fig. 2a,b respectively. The substrate is Rogers RT/duroid 5880 with $\varepsilon _r=2.2$, $\tan \delta =0.0009$, and thickness of $h=1.575$ mm. Other geometrical parameters are $w_g=5.2$ mm, $w_a=25.98$ mm, $l_a=18.5$ mm, $d=0.6$ mm, and $p=1$ mm.

The problem is that when the flare section of SIW horn is made larger, higher order modes can be excited^24^. This deteriorates the performance and a non-uniform aperture with erratic wavefronts is achieved as seen in Fig. 2b which results in increased sidelobes and gain reduction. To address this problem, we aim to transform the generated erratic wavefronts to planar wavefronts to get a better performance. The proposed method can be summarized as first, to realize the required phase compensation pattern and second, to characterise the lens geometry based on the obtained phase requirements.

### Systematic approach to realize the lens location and phase compensation pattern

**Figure 3:**
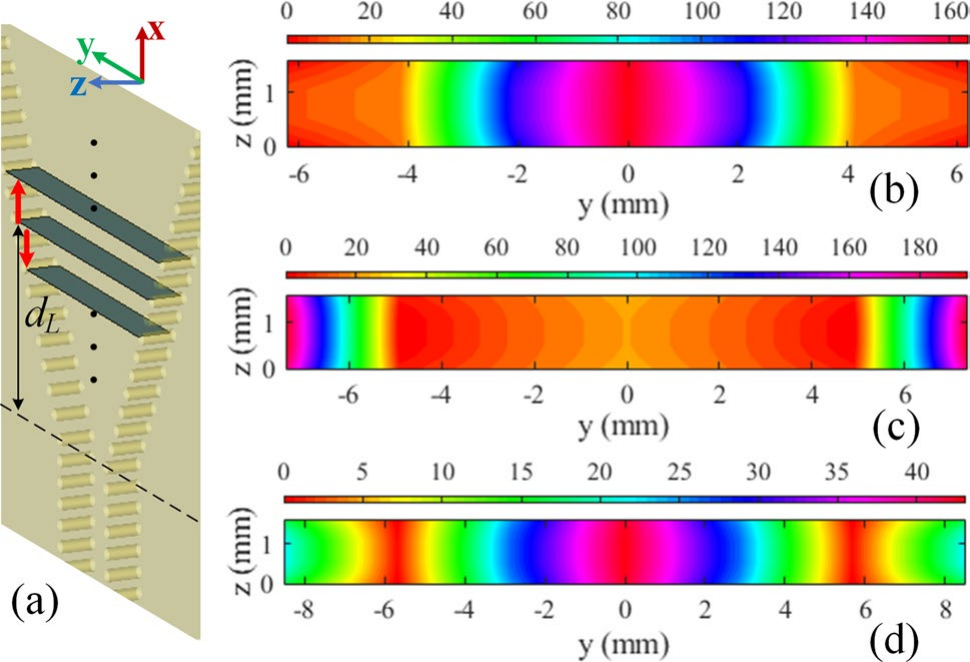
Phase extraction procedure. (**a**) Different cross-sections inside the flare section with variable distances $(d_L)$ from the horn throat. Phase distribution (in degree) at (**b**) $d_L=8$ mm, (**c**) $d_L=10$ mm, and (**d**) $d_L=12$ mm.

Before anything, it should be noted that the lens physical geometry will be inspired by the phase behavior of the fields in the flare section. Therefore, in case of rapid phase variation, implementing the lens would be quite hard, if not impossible. As a result, the first step of realizing the required phase compensation pattern is to find a proper cross-section within the flare in which the local phase would experience a comparably smaller variation. This cross-section would also define the location of lens which is going to be loaded. In comparison with the middle parts of the flare section, the zone close to the opening aperture experiences faster fluctuations of E-field (and phase) which makes it an inappropriate zone to implant the lens. If the lens is located close to the horn throat, as the guided waves move, the phase distribution would be distorted again due to the horn flaring. Considering all these issues, it can be concluded that the ultimate cross-section must be somewhere around the middle of flare section. Let us define $d_L$ as the distance between the horn throat and the discussed cross-section. The aim is to alter the $d_L$, as shown in Fig. 3a, to find a cross-section with the minimum possible range of phase variation. Based on this, we found that the $d_L=12$ mm cross-section has a relatively smaller range of phase variation comparing to other cross-sections in the middle zone of the flare. To illustrate this, the simulated phase pattern for three example values of $d_L=\{8, 10, 12\}$ mm are reported in Fig. 3b–d respectively. Therefore, the $d_L=12$ mm cross-section is chosen to determine the required phase compensation pattern. The lens should be located just behind this cross-section to form a constant aggregated phase at $d_L=12$ mm.

### Characterizing the lens geometry

**Figure 4:**
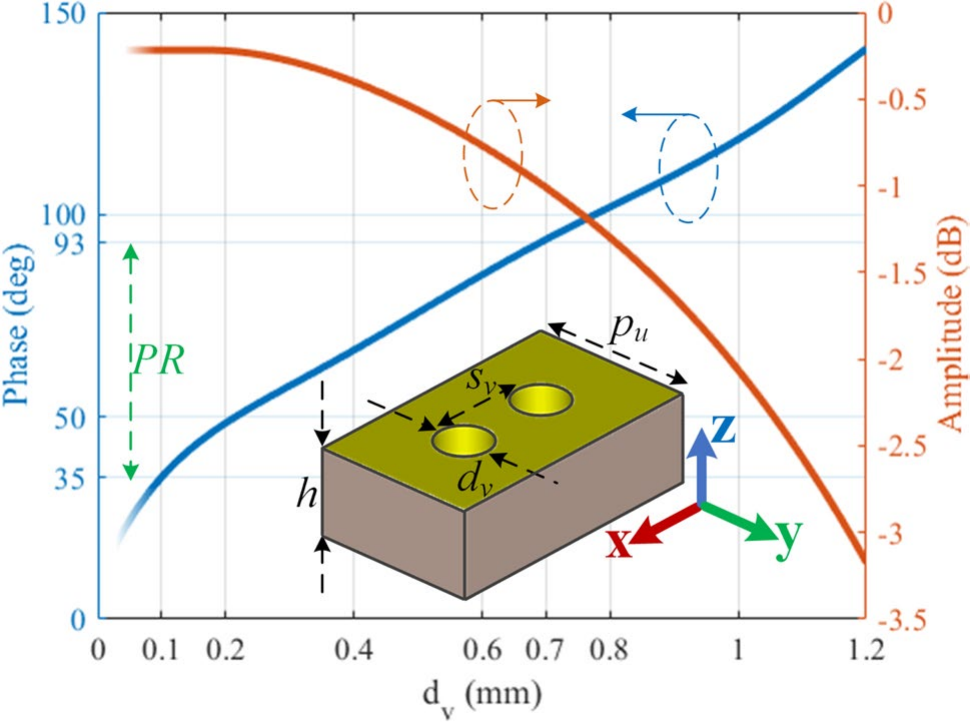
The geometry of the proposed unitcell and its simulated transmission characteristics at 30 GHz. Ports 1 and 2 are configured at the two faces of the unitcell normal to the *x*-axis.

The proposed lens is formed by a set of metallic vias with variable diameter of $d_v$ and fixed lattice period of $p_u= 0.45\times \lambda _g\approx 3$ mm along the transversal axis ( $y$-axis); the unitcell contains two identical vias which are separated by $s_v=\lambda _g/4$ along the longitudinal axis ( $x$-axis) as is presented in Fig. 4. Each single via in a unitcell with its adjacent vias in the neighbor unitcells make a transmission layer altogether. As a rule of thumb, single-layer transmission surfaces have a limited transmission phase range for a standard level of $-\,1$ dB or $-\,3$ dB transmission loss^25^. To enlarge the phase range, the number of layers should be increased. So, the two vias of our proposed unitcell will ultimately make a double-layer transmision surface which can provide sufficient phase range for EM wave manipulation. These vias must be placed behind the selected cross-section of $d_L=12$ mm (see Fig. 3). Therefore, it is not possible to further increase the number of transmission layers (e.g. vias in each unitcell) as it would exceed the physical boundaries of the flare section.

When a wavefront reaches to these vias, it will slide around them and pass through. The diameter of vias will affect the transmitted phase of field, as well as its amplitude. The higher values of $d_v$ will be accompanied by larger phase variation because it will increase the path length that wavefronts are going to cross over. Furthermore, larger values of $d_v$ will result in higher transmission loss to the extent that it can completely block the guided waves. For $d_v \le 1.2$ mm, the transmission characteristics are simulated and displayed in Fig. 4. For $d_v<0.1$ mm, the fabrication process is a challenging task and it is not practical to consider this spot. Ignoring this spot, the achieved phase range is more than $100^{\circ }$ with less than $-3$ dB transmission loss. Note that $d_v>1.18$ mm leads to transmission loss of higher than $-3$ dB.

The next step is to modulate the $d_v$ values based on the calculated localised response of irises (Fig. 4) and by considering the phase distribution of Fig. 3d. The corresponding cross-section (at $d_L=12$ mm) has the length of 17.06 mm towards the *y*-axis. Recalling $p_u=3$ mm, this cross-section can embrace maximum five unitcells. So the sampling points, referring to Fig. 3d are at $y=\{-\,6, -\,3, 0, 3, 6\}$ mm. The phase value at edges of $y=\pm\, 8.53$ mm are $21.93^{\circ }$. Introducing $\Delta$P as the difference between phase at sampling points and edges, the objective is to make $\Delta$P as small as possible to obtain a quasi-uniform phase distribution. The sampling points, their corresponding phase values, and $\Delta$P are presented in Table 1.

As it can be seen from Fig. 4, the transmission loss is less than $-\,1$ dB for $d_v<0.7$ mm with the phase range of $PR=93-35 = 58^{\circ }$ which can cover the whole phase span at the $d_L=12$ mm cross-section (see Fig. 3d). Considering the phase of $60^{\circ }$ as a reference point, the maximum and minimum values of $\Delta$P will still result in phase values within the *PR* zone (see Table 1, Fig. 4). Then, the modulated values of $d_v$ are obtained as listed at the last row of Table 1.

**Table 1:** Modulation procedure of the proposed lens.

*y* (mm)	− 8.53	− 6	− 3	0	3	6	8.53
Phase $_{\text {(deg)}}$	21.93	0.53	26.48	43.1	26.48	0.53	21.93
$\Delta$P $_{\text {(deg)}}$ $^{\dagger }$	N.D.	21.4	− 4.55	− 21.17	− 4.55	21.4	N.D.
$d_v$ (mm)	N.D.	0.56	0.27	0.13	0.27	0.56	N.D.

$$^{\dagger }$$$$\Delta$$P $$=$$ (phase value at edges $${y = \pm \text {8.53}}$$) $${-}$$ (phase at the sampling points).

### Loading implementation, simulation, and measurement results

**Figure 5:**
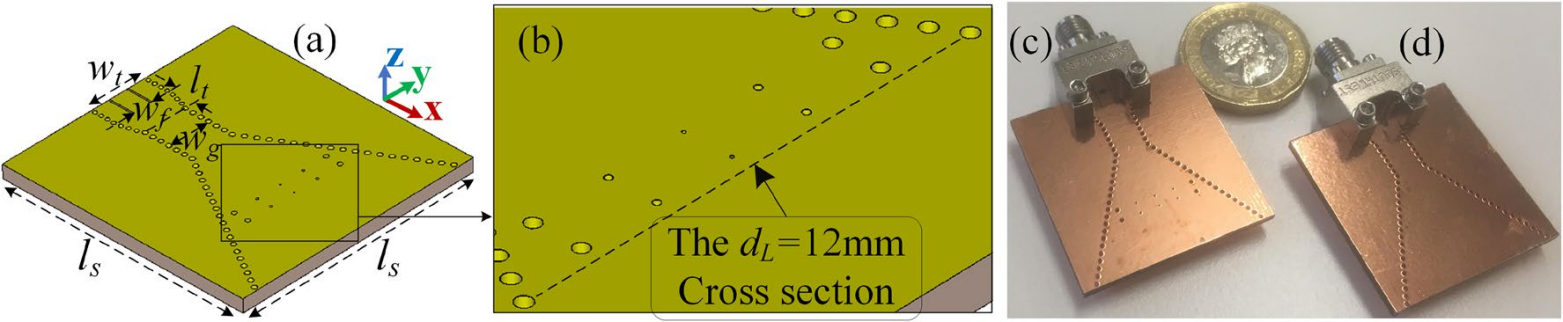
(**a**) Geometry of the proposed SIW H-plane horn loaded by the metal-via lens, (**b**) the lens pattern in a larger scale, (**c**) the fabricated loaded structure, (**d**) the fabricated conventional SIW H-plane horn.

The SIW H-plane horn presented in Fig. 2 is loaded by the designed metal-via lens as shown in Fig. 5a with the substrate size of $l_s=30$ mm. The structure is excited by a grounded coplanar waveguide (GCPW) line with $w_t=6.95$ mm and $w_f=2.2$ mm. In order to make the port matched at the operating frequency, a linear transition with length of $l_t=2.45$ mm is applied from the GCPW line to the guided-wave part with width of $w_g$. The geometry of the proposed lens is magnified in Fig. 5b which is set based on the presented data in Table 1, and placed behind the intended cross-section of $d_L=12$ mm. The proposed loaded and conventional antennas are fabricated and illustrated in Fig. 5c,d respectively where each structure is fed by a 2.92 mm end-launch connector.

**Figure 6:**
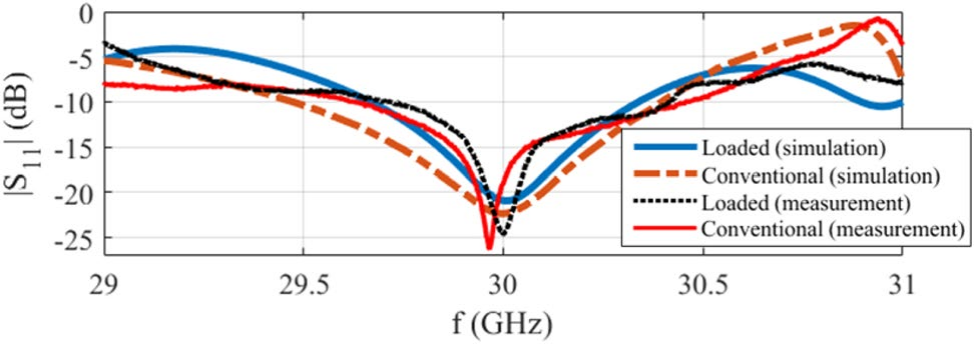
The simulated and measured |S $_{11}|$ of the conventional and loaded structures.

The simulated and measured |S $_{11}|$ of the structures are shown in Fig. 6, stating that the loaded structure is well matched at $f=30$ GHz. It should be noted that when the conventional SIW horn is loaded, the local waves are distorted at the position of the designed lens which subsequently alters the overall impedance response. This shifts the operating frequency and changes the bandwidth as the structure’s response is not only impacted by geometry of the flare and feeding transition, but the embedded lens. To make both conventional and loaded horns operate at the same frequency, the feeding transition should be modified slightly. As a result, the reported S $_{11}$ of the conventional SIW H-plane horn in Fig. 6 is derived with $w_t=6.95$ mm and $l_t=0.95$ mm.

**Figure 7:**
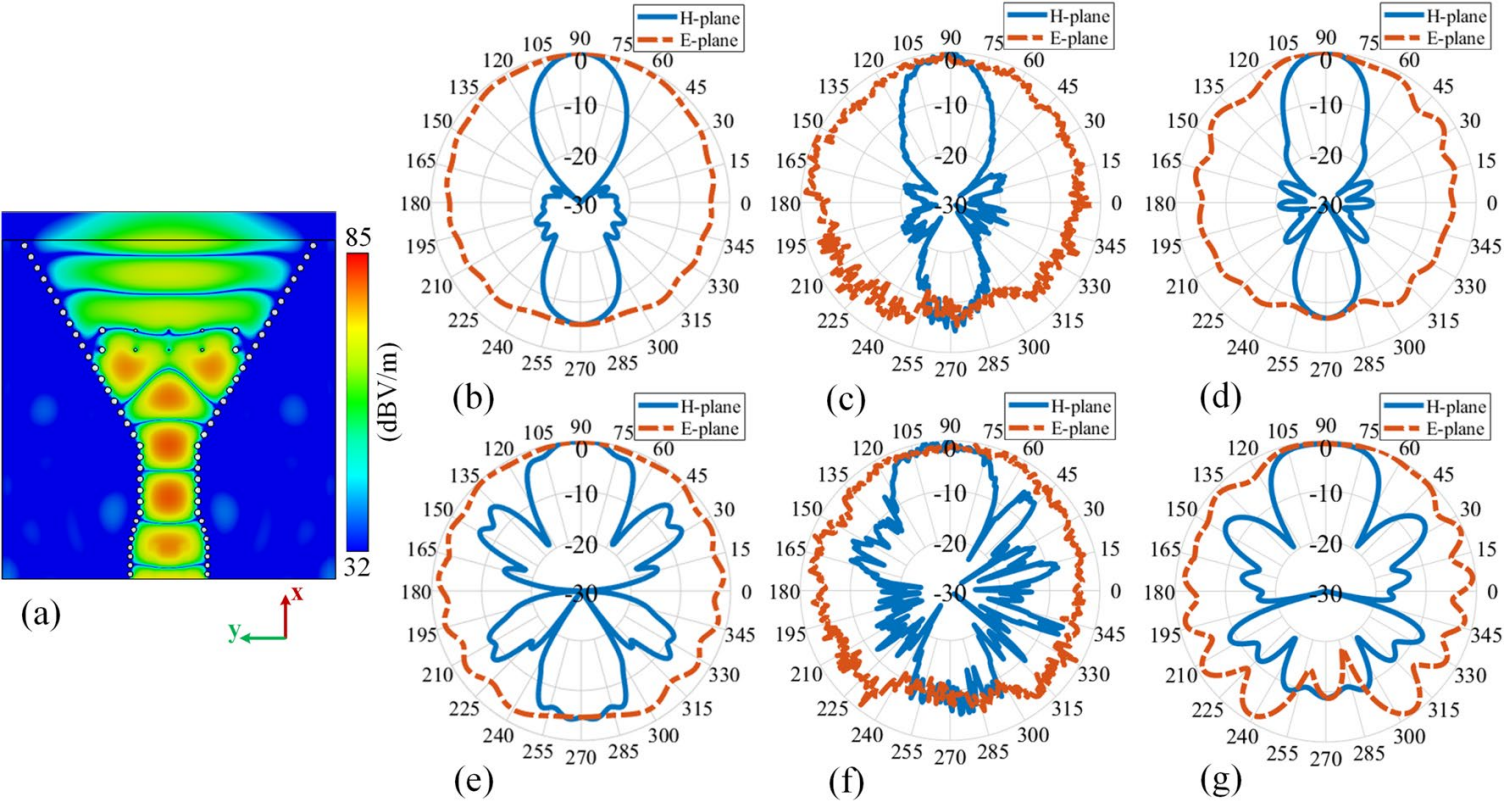
Performance at 30 GHz: (**a**) the simulated |E-field| distribution pattern of the proposed loaded SIW H-plane horn antenna. The normalized radiation pattern: (**b**) simulated loaded structure without the connector, (**c**) measured loaded structure, (**d**) simulated loaded structure with the connector, (**e**) simulated conventional structure without the connector, (**f**) measured conventional structure, (**g**) simulated conventional structure with the connector.

The simulated |E-field| distribution of the proposed phase corrected SIW H-plane horn is shown in Fig. 7a at the operating frequency of $f=30$ GHz. This result clarifies that the distorted wavefront of Fig. 2b is transformed to a planar wavefront when the structure is loaded.

The simulated normalized radiation pattern for both loaded and conventional antennas at $f=30$ GHz are presented in Fig. 7b,e respectively. Based on the simulation results, it is observed that the proposed lens enhances the H-plane radiation characteristics of the conventional antenna in terms of gain (from 5.16 to 8.59 dBi), SLL (from $-\,5.38$ to $-\,19.85$ dB), and F/B (from 4.54 to 5.52 dB).

The measured normalised radiation patterns are shown in Fig. 7c,f for the loaded and conventional structures respectively which are slightly different comparing to the simulated results of Fig. 7b,e. This is due to the presence of the connector which influences the radiation pattern as its physical dimensions are large (concerning the operating frequency of $f=30$ GHz) and is located relatively close to the radiating aperture. Introduction of the connector to the CST environment brings the simulated results (Fig. 7d,g) inline with the measured results (Fig. 7c,f). More details about the connector’s influence will be presented later on in “Discussion” section. The measured gain, SLL, and F/B for the loaded (conventional) antenna are 8.69 (5.21) dBi, $-\,18.06~(-\,5.02)$ dB, and 6.96 (5.89) dB respectively. The counterpart simulated gain, SLL, and F/B for the “connector-included case” regarding the loaded (conventional) structure are 8.72 (5.34) dBi, $-\,18.87~(-\,6.11)$ dB, and 6.89 (8.74) dB respectively.

**Figure 8:**
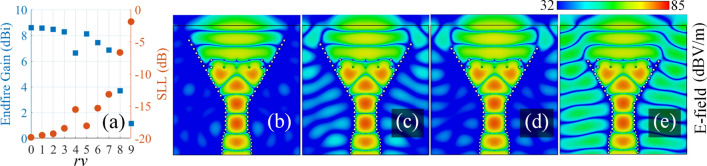
(**a**) The endfire gain and SLL for $rv=0\sim 9$. |E-field| distribution for (**b**) $rv=3$, (**c**) $rv=4$, (**d**) $rv=5$, and (**e**) $rv=8$ at 30 GHz.

Considering the E-field distribution of Fig. 7a, after passing through the lens, the guided waves are mainly concentrated in the middle and are negligible along the edges of the flare section. Therefore, after a distance from the metalized via holes, the two side edges of the horn may need not to be flared anymore. To assess this issue, a series of simulations are carried out. At each simulation, a set of via holes are removed and the performance of the structure is studied. We define *rv* to indicate the number of via hole couples that are removed from the opening aperture. For example, $rv = 1$ $(rv = 4)$ means that the first couple of via holes (the first four couple of via holes) are removed from the opening edge. The obtained endfire gains and SLLs are presented in Fig. 8a for $rv=0$ (the original structure) to $rv=9$. The |E-field| distribution pattern for the cases $rv=\{3,4,5,8\}$ are shown in Fig. 8b–e respectively. Based on Fig. 8a, the constructed gain is not considerably changed for the cases $rv=0{-}3$. This can be interpreted by comparing Figs. 7a and 8b which shows that the field distribution is almost the same in both cases. However, $rv = 4$ is accompanied by a reduction in gain as a destructive leakage is started to happen in the substrate’s body (see Fig. 8c). As *rv* is increased, it is expected to observe that this leakage will also increase; but removing another couple of via holes results in a less leakage as shown in Fig. 8d, so that a local maximum of 8.12 dBi occurs at $rv=5$ (see Fig. 8a). After this specific point, a downward movement is observed for the gain and the leakage dominates as *rv* approaches the lens position (see Fig. 8e for $rv=8$).

Based on this study, it is concluded that the flaring can be stopped as illustrated in Fig. 8d for the case $rv=5$ without expecting a considerable variation on the antenna performance. Hence, the substrate can be transversely cut to have a smaller dimensions. This defines the baseline structure for the next section.

## Holographic-based metal strips: proposed method to recycle the surface waves (step #2)

So far, the radiation properties of the SIW H-plane horn are enhanced by loading the structure with the designed modulated metal-via lens. The flaring effect after a distance from the lens is also studied which can ultimately make the structure transversally smaller. In this section, we aim to improve the radiation characteristics of the antenna further by proposing a new method which is explained hereinafter.

### Definitions and the proposed approach

**Figure 9:**
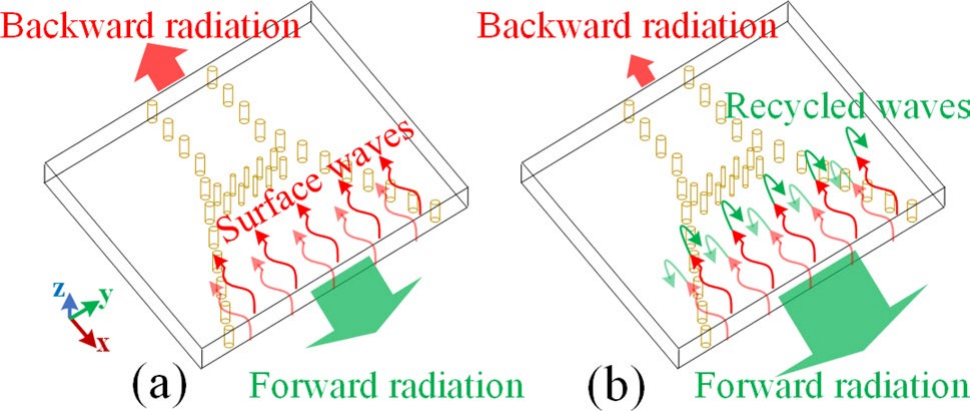
The proposed mechanism for tailoring the radiation characteristics (**a**) the phase corrected SIW H-plane horn, (**b**) the field manipulation method.

As the guided waves reach the radiating aperture, the EM wavefronts expand in the free-space while directing forward. This wavefront expansion into the free-space also causes unwanted backward surface waves in the interface between the metallic broadwall of the SIW H-plane horn and the free space which ultimately increases the backlobe level. This process is illustrated in Fig. 9a. Considering these unwanted surface waves, it is possible to redirect them forward in a way that they can constructively add up to increase the endfire gain and to simultaneously decrease the backward radiation. In other words, the undesired surface waves are recycled to enhance the radiation characteristics of the structure in hand without introducing an additional EM-source. This process is performed by holography technique and is shown in Fig. 9b.

Holography technique, originated from optics, involves generating an interference pattern using two waves, and then utilizing the calculated pattern to scatter one wave to launch the other. The aperture is formed as a result of diffraction of a primary field, which can be a surface wave, by a pattern of scatterers on the structure. The obtained structure is then typically a leaky-wave structure^26^. The primary surface wave is usually generated by a source antenna which is called surface-wave-launcher (SWL), printed on a common substrate where the scatterers are placed. The pattern of scatterers is derived from the holography technique, which enables the surface waves to leak constructively toward the direction of interest. The initial wave from the SWL is called “reference wave”. The intended radiation pattern (leakage direction) is determined by the so-called “object wave”. This term, in its fundamental definition, is a wave that is illuminating the aperture from a hypothetical source located at far distance. The relative position between the far-located source and the aperture is a representative of leakage direction out of the aperture, determining the beam’s tilt angle. This technique composes of two general steps as follows;

- The first step, called as “recording” process, is to calculate the map of reference waves, emitting from the SWL, on the structure. This will specify the phase-line distribution pattern on the substrate with respect to the relevant location and type of the SWL. After that, based on the desired direction of the beam, the superposition of the reference wave and object wave is calculated. This results in another phase-line distribution pattern on the substrate which is called “interfogram” or “EM-hologram”.
- In order to make the structure radiate in the desired direction, it is needed to employ several scatterers on the substrate with a pattern inspired by the recorded EM-hologram. This will build a constructive leakage out of the structure and will form the beam. Based on the structure’s physical and EM characteristics, a proper SWL must be designed and applied to the substrate. This step is called “reconstruction” process.

**Figure 10:**
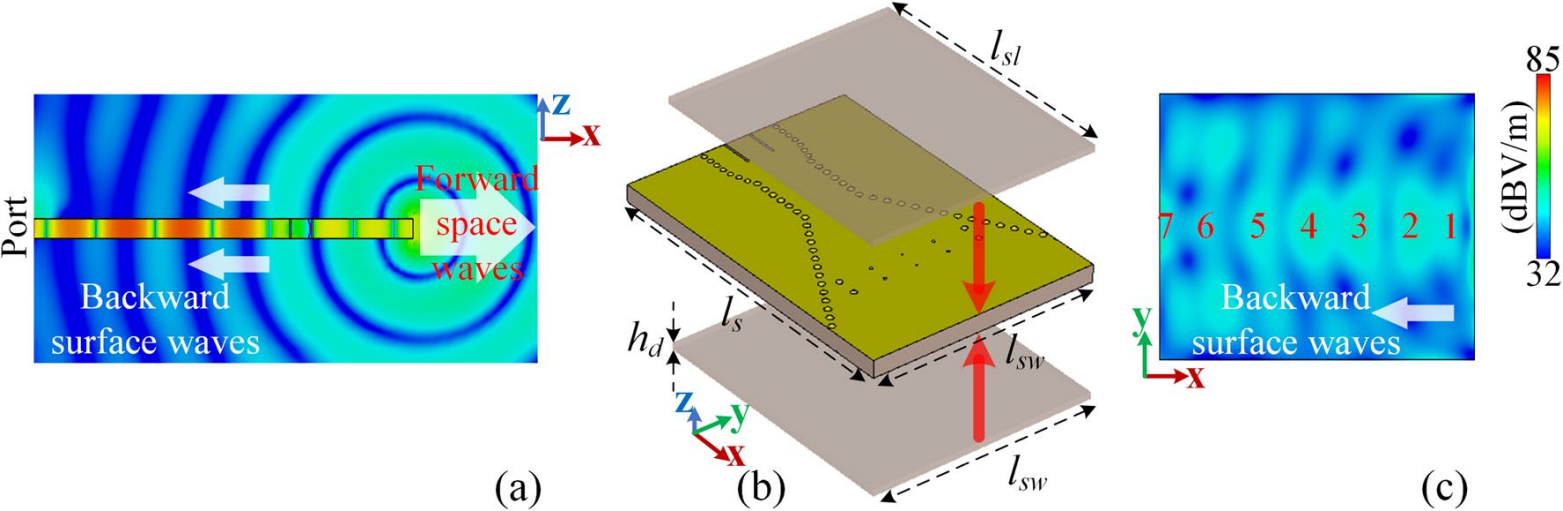
(**a**) The |E-filed| distribution pattern on a cut at *xz* plane crossing the middle of the phase-corrected structure, (**b**) the modified structure with two dielectric slabs of Rogers RT/duroid 6010 attaching to both sides, (**c**) the simulated |E-field| on the slab when it is mounted on the SIW horn. The simulation frequency is 30 GHz.

The scatterers can be periodic metallic patches or complementary slots on a one- or two-dimensional lattice to form a metasurface^27^, dielectric slab with modulated thickness^28^, or continuous metal-strips^29^; we use the last one in this work. Figure 10a shows the |E-filed| distribution pattern on a cut at *xz* plane crossing the middle of the phase-corrected structure. Forward space waves, as well as the backward surface waves can be observed from this figure. To recycle the surface waves, there should be a mechanism to collect them first. So, a couple of dielectric slabs with planar dimensions of $\{l_{sl},l_{sw}\}=\{25,21\}$ mm are attached at both sides of the modified [The flaring is updated based on the study delivered in “Loading implementation, simulation, and measurement results” section] structure’s broad-walls as illustrated in Fig. 10b. The higher permittivity and thickness of slab, the denser E-filed pattern. This E-filed pattern directly regulates the pattern of metal-strip scatterers which will be explained in details later on in this section.

In holography technique, the fidelity of the reconstructed far-field is dependent on the size of the EM-hologram. Here, this size is a prefixed value, limited to the substrate borders of the modified SIW H-plane horn, so there is no flexibility to make it larger. Therefore, the only way to achieve a better response out of the EM-hologram is to make the metal-strip scatterer denser. As a result, Rogers RT/duroid 6010 laminate with $\varepsilon _r=10.7$ is applied for the added slabs. The dielectric slabs are preferred to be as thin as possible to have minimum impact on the final size of the assembled structure. However, as mentioned before, a thicker substrate leads to a denser field distribution pattern which is a desirable factor in our case. So, the thickness of $h_d=0.635$ mm is chosen out of the standard values of RT 6010 laminate to keep the balance between both criteria.

Figure 10c shows the simulated |E-field| on the slab when it is attached on the SIW horn. This quasi-planar field distribution is casting the role of reference wave. Note that in this specific case, there is no individual SWL to excite the reference wave, but the field expansion from the aperture is the actual source to generate it. This will make the proposed work clearly different from the previous holographic-based leaky-wave structures where a feeder must be explicitly designed and dedicated to generate the required reference wave on the guide structure. Considering the effect of metal-via lens, the fields reaching the aperture are uniform which consequently form the presented quasi-planar reference waves on the slab.

### Recording process

**Figure 11:**
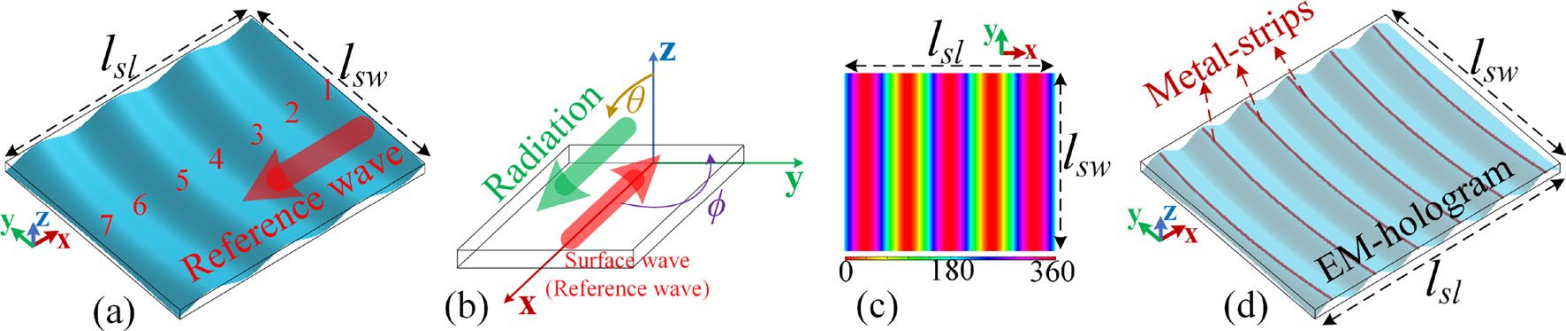
Applying holography technique on the Rogers RT/duroid 6010 dielectric slab; (**a**) the quasi-planar reference wave on the structure, (**b**) the dielectric slab and the direction of intended radiation in a standard right-handed coordinate system, (**c**) object wave’s phase lines map on the structure, (**d**) calculated EM-hologram and the corresponding metal-strips pattern, named as the surface-waves recycler (SWR).

To derive the EM-hologram, it is necessary to define an analytical expression of reference wave, $E_{\text {ref}}$, on the dielectric slab at the first round of calculation. For a planar wavefront which is surfing the dielectric slab toward $-x$ with phase constant of $\beta _{\text {ref}}=2\pi /\lambda _{\text {ref}}$ and amplitude of *A*, the reference wave is $E_{\text {ref}}=A e^{j\beta _{\text {ref}} x}$. But, regarding Fig. 10c, the coupled surface waves on the dielectric slab are not purely planar; for an EM-hologram, the very first consideration that is required to be taken into account is to estimate the $E_{\text {ref}}$ pattern as much accurate as it is possible. In this case, a more expressive formulation of the reference wave can be defined as below:

2 $$\begin{aligned} E_{\text {ref}}=A e^{-j\beta _{\text {ref}} r_m}, \end{aligned}$$

with $r_m=\sqrt{w_x (x-c_x)^2 + w_y (y-c_y)^2}$, indicating the modified radial distance on the *xy* plane (where the slab lays on). This formulation is emulating a point-source located at $(x=x_c, y=y_c)$ which will generate sort of radial traveling waves on the *xy* plane. The idea is to put this point-source far from the slab, then, the radial waves can be tuned by the appropriate weighting of $w_x$ and $w_y$ in a way that a portion of total propagation plane on the dimensions of the slab properly mimics the pattern of Fig. 10c. The weighting factors are determining how fast the emitted waves from the point-source would vary along *x* and *y* axis; so, a variety of 2D patterns can be formed by altering $w_x$ or $w_y$. This ends up with $\{w_x, w_y, c_x, c_y\}=\{0.4, 1, 0.275, 0\}$ in our case study where *x* and *y* are in meter. The obtained pattern of $E_{\text {ref}}$ on the dielectric slab is presented in Fig. 11a which is in line with Fig. 10c.

The next step is to capture the phase-front of the object wave, $E_{\text {obj}}$, on the dielectric sheet. Figure 11b shows the dielectric slab in a standard right-handed coordinate system with the direction of the intended beam at $(\theta _m=\pi /2, \phi _m=0)$. Assuming an object wave along the desired beam direction which is illuminating the dielectric slab, the map of $E_{\text {obj}}$ on the slab is obtained using the following equation:

3 $$\begin{aligned} E_{\text {obj}}(x,y)=B e^{j k_0 \{\sin {(\theta _m)}\cos {(\phi _m)}x+\sin {(\theta _m)}\sin {(\phi _m)}y\}}, \end{aligned}$$

where *B* is the amplitude and $k_0$ is the wave vector of the space waves. Applying this equation and considering the beam direction, the obtained phase-front pattern is presented in Fig. 11c.

As the final step, the superposition of Eqs. (2) and (3) leads to an interference pattern which is defining the EM-hologram as illustrated in Fig. 11d.

### Reconstruction process and hologram realization

As no individual SWL is required for this specific design, the reconstruction process is summarized by applying continuous metal-strips at the local maxima of the calculated interference pattern as specified in Fig. 11d. These strips shorten the E-field lines of the surface wave at their positions which consequently form the roots of the interference pattern that can make the beam. Concerning Fig. 9b, let us call the EM-hologram, together with the corresponding metal-strips, the “surface-waves recycler” (SWR).

**Figure 12:**
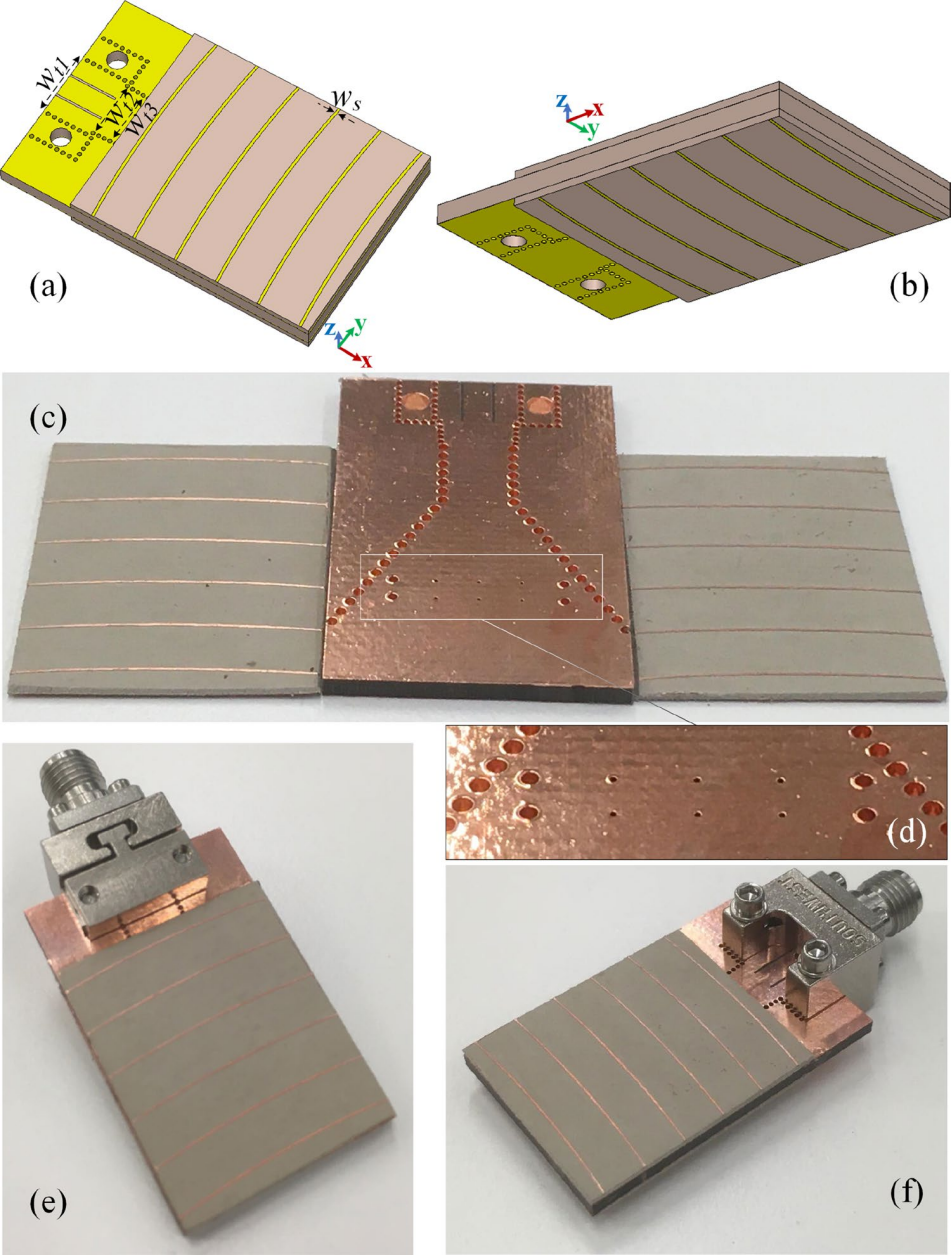
The finalised structure. (**a**) The topside and (**b**) the backside of the simulated structure, (**c**) the fabricated SIW H-plane horn, loaded by the designed modulated metal-via lens, beside the two holographic-based SWRs, (**d**) the magnified view of the modulated metal-via lens, (**e**) the assembled structure from the backside angle of view and (**f**) from the top side angle of view.

In order to realize the SWR and make the hologram deliver its best possible response, the strip’s width must be chosen properly. A too narrow strip cannot be *felt* by the guided waves inside the slab while a too wide one will make the pattern of reference-wave distorted to the extent that the subsequent processes would no longer be valid. Based on our simulation results, $w_s=0.25$ mm is found as the optimum value of strip’s width. Therefore, a couple of hologram sheets with the strips pattern of Fig. 11d and the strip’s width of $w_s$ are mounted on both sides of the designed SIW H-plane horn, making the finalized structure as presented in Fig. 12. Other geometrical parameters of the structure (see Fig. 12a) are $w_{t1}=6.6$ mm, $w_{t2}=5.8$ mm, and $w_{t3}=5.6$ mm. The fabricated SIW H-plane horn with the two holographic-based SWRs are shown in Fig. 12c with a zoomed view on the modulated metal-via lens in Fig. 12d. The structure is fed by a 2.92 mm end-launch connector as shown in Fig. 12e,f.

## Performance assessment

**Figure 13:**
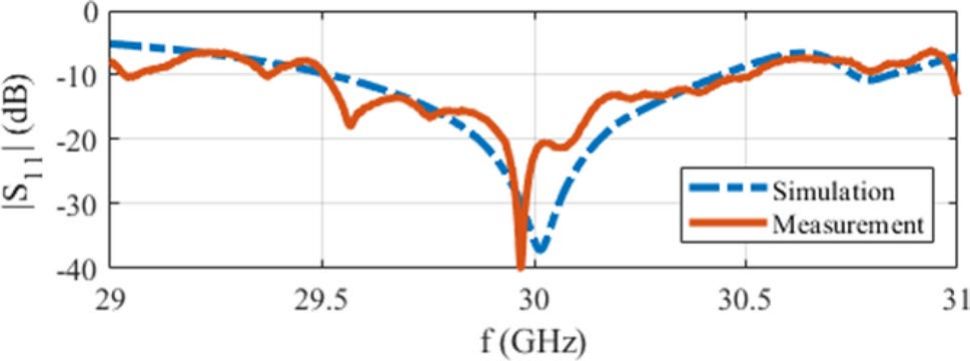
The simulated and measured |S $_{11}|$ of the final assembled design.

Considering the final assembled structure, the simulated and measured reflection coefficients are presented in Fig. 13, stating that the structure is matched well at the operating frequency of $f=30$ GHz. The slight difference between the simulation and measurement results is mainly due to the fabrication imperfections, in particular regarding the bonding between the multiple layers of the fabricated prototype. Especially at high frequencies investigated in this work, the fabrication tolerances can be extremely tight, and such small-scale defects can affect the antenna response.

**Figure 14:**
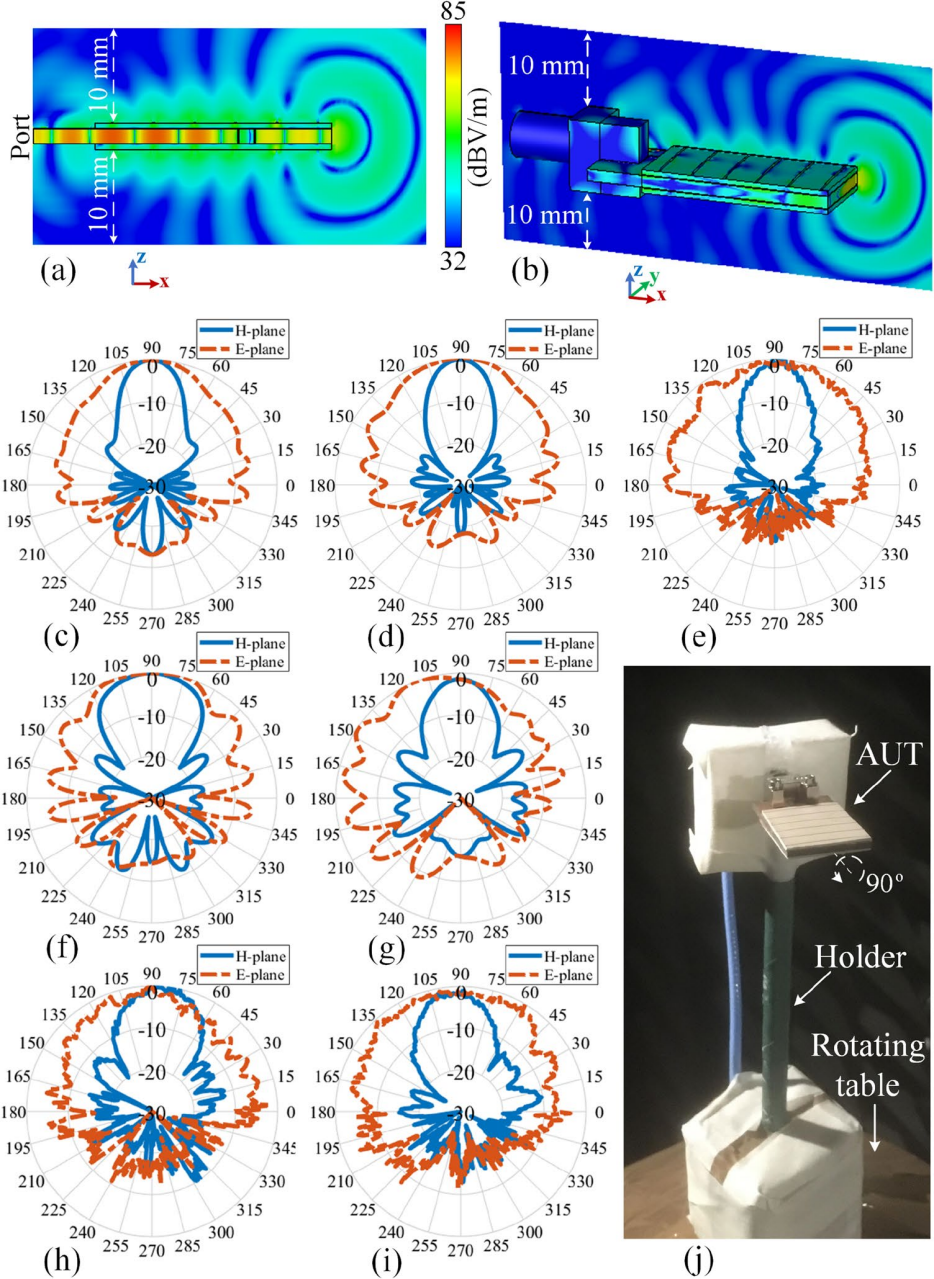
Performance of the final assembled prototype. The simulated |E-filed| distribution pattern at 30 GHz on the *xz* plane cut which is crossing the middle of the structure for the case of (**a**) without and (**b**) with the connector. The normalized radiation pattern at 30 GHz: (**c**) simulated without the connector, (**d**) simulated with the connector, (**e**) measured. The normalized simulated radiation pattern (with considering the connector) at (**f**) 29.5 GHz and (**g**) 30.5 GHz. The counterpart measured results at (h) 29.5 GHz and (**i**) 30.5 GHz. (**j**) The antenna under test (AUT) in an anechoic chamber (H-plane radiation pattern measurement).

The simulated |E-filed| distribution pattern on the *xz* plane cut (crossing the middle of structure) is shown in Fig. 14a. With this figure, it can be clearly observed that the backward surface waves of Fig. 10a are now properly manipulated and curved to the forward direction. In order to have a more realistic analysis, the connector is included in the simulation environment. This results in Fig. 14b where the field is no longer symmetrically distributed with respect to the *x*-axis in comparison with Fig. 14a. This means that the corresponding radiation pattern cannot be expected to be symmetrical in the E-plane. More importantly, the connector is clearly suppressing the intensity of backward wave which will consequently decrease the backlobe radiation. Hence, the connector brings a constructive influence on the radiation characteristics in our specific case. The normalized simulated radiation pattern without considering the connector is presented in Fig. 14c at the frequency of $f=30$ GHz with the gain, SLL, and F/B of 11.23 dBi, $-17.02$ dB, and 13.21 dB respectively. The counterpart result for the case of “taking the connector into account” is shown in Fig. 14d with the corresponding gain, SLL, and F/B of 11.71 dBi, $-\,18.35$ dB, and 18.16 dB respectively. The measured radiation pattern at $f=30$ GHz is presented in Fig. 14e with the obtained gain, SLL, and F/B of 11.65 dBi, $-17.94$ dB, and 17.02 dB respectively. The results show an obvious increment (decrement) of the forward (backward) radiation comparing to what is reported in “Loading implementation, simulation, and measurement results” section. This suggests that in comparison to the conventional SIW H-plane horn, the measured gain and F/B are increased by 6.44 dB and 11.13 dB respectively while the SLL is reduced by 12.92 dB. The simulated radiation patterns of the structure at $f=29.5$ GHz and $f=30.5$ GHz are shown in Fig. 14f,g with the gain of 9.33 dBi and 10.52 dBi respectively. The counterpart measured results are presented in Fig. 14h,i with the respective gain of 9.15 dBi and 10.24 dBi. As the attached EM-holograms are leaky-wave structures, their responses are frequency dependent. This means that the direction of the leaked beams will be changed by sweeping the frequency. The hologram pattern is calculated at $f=30$ GHz, hence, the structure shows its best performance at this frequency as the constructed beams are highly aligned with the object beam at this frequency.

## Methods

Figure 14j shows the antenna under test (AUT) in an anechoic chamber in the setup of measuring the H-plane radiation pattern. The measurement procedure is to rotate the AUT around its holder’s axis while being illuminated by a reference antenna (a horn antenna with known characteristics in our case). The system is then calibrated and the received power is captured at every single angle of rotation to derive the plot of radiation pattern over the entire range of angles. The same steps are repeated to read the E-plane radiation pattern with the AUT rotated by $90^{\circ }$ around its longitudinal axis as indicated in Fig. 14j.

## Discussion

To have a clear picture of the influence of each manipulating component, the corresponding radiation characteristics at the presence of each component are summarized in Table 2. This shows that when the holographic metal strips are added to the phase corrected structure (loaded by modulated metal via lens), the gain and F/B are obviously enhanced, but the side lobes are slightly grown. This is due to the redirection of SWs on the slabs which affects both the main and side lobes. However, the obtained final SLL is still far better than the original structure.

**Table 2:** The effect of adding manipulating components to the original structure on the radiation characteristics.

	Gain (dBi)	SLL (dB)	F/B (dB)
Original structure	5.34 (5.21)*	− 6.11 (− 5.02)	8.74 (5.89)
+ Modulated metal via lens	8.72 (8.69)	− 18.87 (− 18.06)	6.89 (6.96)
+ Holographic-based metal strips	11.71 (11.65)	− 18.35 (− 17.94)	18.16 (17.02)

*The simulated (measured) results at $f=30$ GHz.

As mentioned before, the size of the hologram is an important factor to get a proper response out of it. This means the bigger the substrate’s sheet, the better-shaped the reconstructed beam. In our specific case, the size of the holograms are relatively small (as they are confined to the physical margins of the SIW H-plane horn), but, the radiation metrics clearly showcase that the presence of the designed SWRs are productive enough to enhance the radiation characteristics.

A comparison study between the proposed work with the state-of-the-art on manipulated SIW H-plane horn antennas is presented in Table 3. Note that in some of these works, an array is formed from the designed elements. To have a meaningful comparison with other works, the reported data in those cases are corresponded to the performance of the relevant single element. Based on this study, our employed hybrid technique can practically offer the following upside points over the other works; with a moderate level of manufacturing complexity, summarizing as to print three boards and bond them together, the proposed manipulating technique is the only case that improves the gain, SLL, and F/B altogether. The achieved combined gain is higher than all other SIW horns that construct a fan-beam radiation pattern. A high gain antenna is proposed in Ref.^16^, however that structure is designed to form a sharp pencil beam (the aperture is shaped in a 2D format where the thickness of structure is expanded by almost six times) which is not comparable with our proposed fan beam antenna. Considering the difference between the initial and final radiation characteristics, the SLL reduces the most in the proposed structure compared to other works.

**Table 3:** Comparison Study of the proposed structure with the state-of-the-art.

Ref	Loading influence (before $\rightarrow$ after)				Manipulating component	Beam type	Improved performance	Manufacturing complexity
	Gain (dBi)	SLL (dB)	F/B (dB)	Dimensions $^{*}$				
^4^	5.75→	− 11.78→	6→	2 $\times$ 1.3 $\times$ 0.22→	Dielectric slab	Fan	Gain, F/B	Low
	9.7	− 8	20	2.9 $\times$1.3 $\times$0.22				
^6^	N.A.→	N.A.→	N.A.→	2 $\times$1.4 $\times$0.24→	Dielectric slab + printed patch	Fan	SLL, F/B	Low
	10.1	− 12	17	3 $\times$1.4 $\times$0.24				
^7^	N.A.^†^	− 7→	2.2→	1.5 $\times$1.2 $\times$0.1→	Slots on flare	Fan	F/B	Low
		− 12	21.6	1.5 $\times$ 1.2 $\times$ 0.1				
^10^	5.58→	N.A.→	N.A.→	3.5 $\times$ 1.9 $\times$ 0.18→	Metal-via array	Fan	Gain	Moderate
	7.87	− 9.53	4	3.5 $\times$1.9 $\times$0.18				
^13^	N.A.→	N.A.→	N.A.→	5.4 $\times$2.2 $\times h\rightarrow$	Empty SIW	Fan	Gain	Moderate
	7.96	− 8.55	37	5.4 $\times$2.2 $\times$4*h*				
^16^	6.75→	− 22→	4.8→	1.8 $\times$1.5 $\times$0.1→	Waveguide + dipole array + reflector nails	Pencil	Gain, F/B	High
	13.97	− 22	17–24	2.3 $\times$1.5 $\times$0.6				
This work	5.21→	− 5.02→	5.89→	3 $\times$ 3 $\times$ 0.15→	Modulated metal-via + holographic metal strips	Fan	Gain, SLL, F/B	Moderate
	11.65	− 17.94	17.02	3 $\times$ 2.1 $\times$ 0.28				

*The reported values are the maximum edge-to-edge sizes of structure in the format of “$\text {length}/\lambda _\text {o} \times \text {width}/\lambda _\text {o} \times \text {height}/\lambda _\text {o}$” with $\lambda _\text {o}$ is the free space wavelength regarding the frequency at which the structure shows its optimum performance.

$^{\dagger }$The reported value is for the corresponding 1 $\times$4 array which is 10.4 dBi.

## Conclusions

By manipulating the electromagnetic waves inside the flare section of a SIW H-plane horn, a phase corrected structure is obtained. The proposed method is to regulate the phase at specified sampling points by using a set of unit cells. Each unit cell contains two irises distanced by $\lambda _g/4$ across the longitudinal axis, while the diameter of vias are modulated and exhibiting different values as we move along the transversal axis. These vias form a lens altogether which leads to a radiating aperture benefiting from a quasi-uniform phase distribution, getting a better performance in terms of gain and SLL.

After that, a holographic-based method is presented to utilize the backward surface waves on the broad walls of the SIW H-plane horn and direct them forward to enhance the gain even further and to decrease the back-lobe. To realise this, a pattern of metallic strips is derived and printed on a couple of dielectric slabs which then are mounted on both broad walls.

Cascading the above-mentioned procedures makes a hybrid technique capable of simultaneously improving the three radiation characteristics of gain, SLL, and F/B. The structure is designed, fabricated, and tested where shows its best performance at the intended frequency of 30 GHz over its 1 GHz bandwidth.

## Data Availability

Te data that support the findings of this study are available from the corresponding author upon reasonable request.
